# Research on the drought tolerance mechanism of *Pennisetum glaucum* (L.) in the root during the seedling stage

**DOI:** 10.1186/s12864-021-07888-5

**Published:** 2021-07-23

**Authors:** Ailing Zhang, Yang Ji, Min Sun, Chuang Lin, Puding Zhou, Juncai Ren, Dan Luo, Xiaoshan Wang, Congyu Ma, Xinquan Zhang, Guangyan Feng, Gang Nie, Linkai Huang

**Affiliations:** 1 College of Grassland Science and Technology, Sichuan Agricultural University, Chengdu, 611130 China; 2 Sichuan Animal Science Academy, Chengdu, 610066 China; 3 College of Animal Science and Technology, Southwest University, Rongchang Campus, Chongqing, 402460 China

**Keywords:** Pearl millet, Root, Transcriptome, MAPK signaling pathway, Plant hormone signal transduction, ABA

## Abstract

**Background:**

Drought is one of the major environmental stresses resulting in a huge reduction in crop growth and biomass production. Pearl millet (*Pennisetum glaucum* L.) has excellent drought tolerance, and it could be used as a model plant to study drought resistance. The root is a very crucial part of plant that plays important roles in plant growth and development, which makes it a focus of research.

**Results:**

In this study, we explored the mechanism of drought tolerance of pearl millet by comparing physiological and transcriptomic data under normal condition and drought treatment at three time points (1 h, 3 h and 7 h) in the root during the seedling stage. The relative electrical conductivity went up from 1 h to 7 h in both control and drought treatment groups while the content of malondialdehyde decreased. A total of 2004, 1538 and 605 differentially expressed genes were found at 1 h, 3 h and 7 h respectively and 12 genes showed up-regulation at all time points. Some of these differentially expressed genes were significantly enriched into ‘metabolic processes’, ‘MAPK signaling pathway’ and ‘plant hormone signal transduction’ such as the ABA signal transduction pathway in GO and KEGG enrichment analysis.

**Conclusions:**

Pearl millet was found to have a quick drought response, which may occur before 1 h that contributes to its tolerance against drought stress. These results can provide a theoretical basis to enhance the drought resistance in other plant species.

**Supplementary Information:**

The online version contains supplementary material available at 10.1186/s12864-021-07888-5.

## Introduction

Drought is one of the major environment constraints that limits agricultural production worldwide and leads to the lack of adequate moisture that is required for normal plant growth and development and to complete their life cycle [1–6]. Drought stress severely affects the plants by causing substantial reductions in the crop growth and biomass accumulation. The main consequences of drought stress in plants are the reduced rate of cell division and expansion, root proliferation, stem elongation and leaf size. Drought also disturbs the stomatal oscillations, plant water and nutrient relations that result in declining the crop productivity, and water use efficiency [7–9]. It has been reported that drought imposed negative influence on many crops. For example, rice (*Oryza sativa* L.) suffered a drastic yield reduction range of 18-60% and even more than 70% in some places due to water deficiency [10–15] while it caused a 10-50% reduction in wheat (*Triticum aestivum* L.) [16–18]. Moreover, the biomass of maize (*Zea mays* L.) decreased by 1-76% [19–21] and barley (*Hordeum vulgare* L.) by 73-87% upon drought stress, respectively [22]. In addition, the leguminous crops like chickpea (*Cicer arietinum* Linn.), pigeon pea (*Cajanus cajan* (Linn.) Millsp.) and canola (*Brassica napus* L.) planted on arid lands had faced severe reduction in their yield because of drought conditions [23]. These reports indicate that drought stress can lead to an economic impact which will depress the living quality of humans [23]. More significantly, many researchers predicted that arid land would expand globally by the end of this century and was 5.8 × 10^6^ km^2^ (or approximately 10%) larger than that between 1961and 1990 because of increasing concentrations of greenhouse gases in the atmosphere. In addition, the major expansion of arid regions will occur over southwest North America, southern Africa, the northern fringe of Africa, and Australia, while major expansions of semiarid regions will occur across southern Africa, North and South America and the north side of the Mediterranean [24–26]. Therefore, it is crucial to enhance the drought stress tolerance in corps.

Pearl millet (*Pennisetum glaucum* (L.) R. Br.), as the sixth most important economical cereal crops after rice, wheat, maize, barley and sorghum (*Sorghum bicolor* (L.) Moench) in the world [27–34], is cultivated on ~ 27 million hectares worldwide as a staple food crop in arid and semi-arid regions of sub-Saharan Africa, India and South Asia where grain yields average 900 kg/ha [35, 36]. This crop feeds more than 90 million farmers that live in poverty and is highly nutritious (8–19% protein), high in fiber (1.2 g/100 g), low in starch, and has higher concentrations of micronutrients (iron and zinc) than wheat, rice, sorghum and maize [36]. Its planting on the dryland often results in the excellent drought resistance of pearl millet while simultaneously, it is tolerant to heat, salinity and deficiencies in soil nutrient [35, 37, 38]. Studying the mechanisms of drought resistance in pearl millet and mining the key genes related to drought tolerance are very important for pearl millet to acclimatize in severe water deficit environment in the future, which can decrease economic losses, particularly for those areas where pearl millet is used as a main staple food. In addition, it is also beneficial as a source of genetic improvement to raise drought tolerance of other crops.

The root is one of the most important tissues of plants for water uptake and transport and very sensitive to water deficiency [39]. In addition, a main challenge in developing drought-resistant plants is elucidating how roots can better meet the increased evapotranspiration demands of canopy with lower soil water availability, which indicates that studying drought tolerance in root is an important goal. Alternatively, plant establishment at the seedling stage decides its quality for later growth [40]. A transcriptomic analysis under next generation sequencing (NGS) is an efficient approach for exploring gene expression profiling. In addition, RNA-Seq based on NGS has been utilized as a comprehensive high-throughput approach to reveal the variation in gene expression, regulatory networks, and some technology developments in various species [41–46]. In this study, RNA-Seq was performed to detect the seedling stage pattern of expression of roots at early phases, such as the seedling stage, with three time points (1 h, 3 h and 7 h) after drought stress. Currently, three studies have investigated drought resistance in pearl millet using transcriptomic methods [41, 47, 48]. However, none of them conducted an examination of varied gene expression under drought stress over a time course, which is significant because at different times, there may be variations in the expression of gene patterns.

In this study, we analyzed genes that were differentially expressed after drought treatment at different time points (1 h, 3 h and 7 h) in the roots of pearl millet at seedling stage. By analyzing these transcriptome data, we aimed to reveal the early dynamic molecular regulation of pearl millet subjected to drought stress and elucidate the key genes that are responsible for the drought tolerance. This is also important for the drought tolerance of other crops. To the best of our knowledge, there is no information about the early dynamic mechanisms of drought response in pearl millet roots.

## Materials and methods

### Plant growth and water treatment

A cultivar of pearl millet ‘Tifleaf 3’ (provided by Beijing Mammoth Seed Company) was used in this experiment. Twenty plastic pots (10*15 cm) were filled with half silica sand where 0.2 g seeds (about 240 seeds) were spread on the silica sand for each pot. The materials were subjected to grow in the growth chamber which was set a day (14 h)/night (10 h) and temperature regime of 26/22 °C. In the first 3 days, these materials were watered with distilled water and most of them (about 85%) sprouted on the third day. From the fourth day, they were watered with Hoagland nutrient solution (0.5×). After 13 days of growth (most of plants with three leaves), the Hoagland solution of 10 pots was changed as 20% PEG (polyethylene glycol 6000) solution (dissolve PEG in Hoagland solution) which could simulate drought stress [49]. Root samples with similar growth vigour of plants were collected randomly after 1, 3 and 7 h after treatment containing treatment groups and control groups. With 3 biological replicates each and were frozen immediately in liquid nitrogen and stored at − 80 °C for further experiment.

### Physiological index measurement

#### Measurement of the relative electrical conductivity (REC)

0.1 g of pearl millet root tissue was taken and wrapped with gauze, then it was put in a 50 mL centrifuge tube containing 20 mL of deionized water. After 12 h, the first electrical conductivity was recorded as S1. Next, the centrifuge tube was put into boiling water for 15 min, cooled at room temperature with tap water, and used to measure second electrical conductivity recorded as S2. Calculation of the relative electrical conductivity of the root was using the following formula:

$$ \mathrm{REC}=\mathrm{S}1/\mathrm{S}2 $$

#### Measurement of malondialdehyde content (MDA)

The content of malondialdehyde (MDA) was determined by the thiobarbituric acid method [50]. The crude enzyme solution was extracted by taking about 0.1 g of pearl millet root tissue and adding 1.5 mL of phosphate buffer for homogenization in an ice bath. Then, the mixture was centrifuged at 12000 g at 4 °C for 15 min and the supernatant was the crude enzyme solution. The protein content of the crude enzyme solution was calculated through the protein standard curve. Next, 1 mL reaction solution (including 20% trichloroacetic acid and 0.5% thiobarbituric acid) was added into 0.5 mL crude enzyme solution and the mixture was put in 95 °C water bath for 15 min. After cooling, the centrifuge tube was centrifuged at 12000 g at 25 °C for 10 min. Finally, the absorbance of supernatant was measured at 532 nm and 600 nm, respectively, and recorded as A532 and A600, then ΔA = A532-A600. The content of MDA was calculated by using the following formula:

$$ \mathrm{MDA}\ \mathrm{concentration}:\mathrm{C}\left(\mathrm{mmol}/\mathrm{L}\right)=\Delta \mathrm{A}/\left(\mathrm{l}\times \varepsilon \right) $$

$$ \mathrm{MDA}\ \mathrm{content}\ \left(\mathrm{mmol}/\mathrm{mg}\right)=\mathrm{C}\times \mathrm{V}\times {10}^{-3}/\mathrm{Cpr} $$

Note: Among them, l: 96-well plate optical path, 0.5 cm; ε: extinction coefficient 155 mM^− 1^ cm^− 1^; C: MDA concentration (mmol/L); V: total volume of extracted crude enzyme solution (mL); Cpr: material protein content (mg).

### RNA-seq and data analysis

RNeasy Plant Mini Kit was used to extract RNA of samples and the quality of RNA was examined by RNA gel electrophoresis. A NanoDrop spectrophotometer (California, USA) was used to detect the purity of RNA, and a Qubit RNA assay kit in a Qubit 2.0 fluorometer system (California, USA) was used to determine the concentration of RNA. The library was constructed by the NEBNext® UltraTM Directional RNA Library Prep Kit for Illumina® (California, USA). The mRNA was enriched by The NEBNext®Poly (A) mRNA Magnetic Isolation Module while Fragmentation Buffer was used to break mRNA into short segments. A strand of cDNA was synthesized with random hexamer primers and the second strand was synthesized by adding buffer, DNA polymerase I and dNTPs. Both strands of cDNA were purified by AMPure XP beads, which was repaired at the end. A tail was added and sequenced. Then, fragment size was selected by AMPure XP beads. At last, the final cDNA library was gained by PCR enrichment. Qubit 2.0 was used for preliminary quantification and Agilent 2100 was used to test the inserted fragments of the library [29]. Furthermore, Illumina Hi-Seq 2000 was used for sequencing. We established a total of 18 RNA-Seq libraries.

Identification of gene expression level of each sample was carried out by using the Kallisto software [51]. The clean data produced by Illumina sequencing were mapped to Pacbio sequencing data (SRR11816223) of pearl 

millet [29], and the read count of each gene was gained from the mapping results [52]. The read count value of each gene was converted to the FPKM value (Fragments per Kilobase Million).

Differential expression analysis of two groups (control and drought treatment groups at each time points) was performed by using software and the DESeq2 [53] and genes with an adjusted *P*-value < 0.05 and |log_2_ (FC)| ≥ 1 found by DESeq2 were assigned as differentially expressed. Gene Ontology (GO) enrichment analysis of differentially expressed genes (DEGs) obtained was implemented by the GOseq R package. Besides, GO terms with corrected *P*-value less than 0.05 were thought enriched significantly. Finally, the KOBAS 3.0 was used to test the statistical enrichment of DEGs in KEGG pathways [29].

A weighted gene co-expression network analysis (WGCNA) was carried out by the WGCNA package in R (v3.3.0) (https://horvath.genetics.ucla.edu/html/CoexpressionNetwork/Rpackages/WGCNA/).

## Results and discussion

### Measurement of REC and the content of MDA

**Fig. 1:**
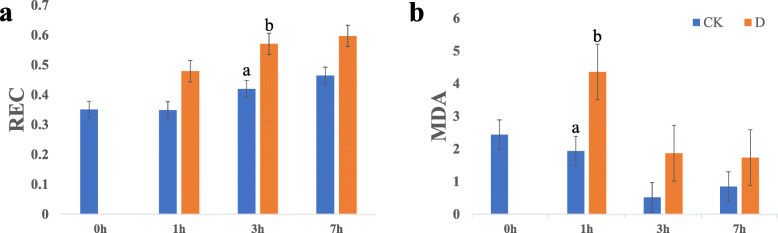
**a** The relative conductivity (REC) at 0 h, 1 h, 3 h and 7 h of CK and drought group in root. **b** the content of malondialdehyde (MDA) at 0 h, 1 h, 3 h and 7 h of CK and drought group in root

The pearl millet root samples were collected after treatments to measure the relative electrical conductivity (REC) and the content of malondialdehyde (MDA). As time increased, the REC went up in both CK and drought stress groups (Fig. 1). After a short period of exposure to drought, the REC of the roots was higher than when it was grown in normal conditions and expressed a significant difference at 3 h. In contrast, the content of MDA declined from 0 h to 3 h in the CK group, after that it was slightly increased. Moreover, the content of MDA at an early stage (1 h) of drought treatment was significantly higher than that in the CK group and then quickly decreased at 3 h and 7 h.

Under environmental drought stress, reactive oxygen species (ROS) levels increased dramatically, which resulted in severe oxidative damage to DNA, proteins and lipids (Apel and Hirt, 2004). These reactive oxygen species, (such as O^− 2^- and H_2_O_2_, directly attack membrane lipids and increase the peroxidation of lipid [54]. The REC [55] and the content of MDA are considered to be indicators of oxidative damage and MDA is thought to be a marker for membrane lipid peroxidation [56]. A decrease in membrane stability demonstrates the level of lipid peroxidation that is caused by ROS. Moreover, lipid peroxidation can indicate the prevalence of free radical reactions in tissues [9]. Many species such as cucumber (*Cucumis sativus* L.) [55], tobacco (*Nicotiana tabacum* L.) [57], wheat [58] and *Phillyrea angustifolia* L. [59] etc. showed a significant increase in MDA and relative electrical conductivity under drought stress. In our research, the content of MDA showed a significant rise at 1 h that indicates that there might be a substantial production of ROS in a short time after drought treatment that damages proteins and lipids. However, the content of MDA in drought stress group decreased at 3 h and 7 h. This could be due to many proteins like antioxidase in pearl millet were generated so that the ROS were converted to harmless compounds. Therefore, the root showed a decline in MDA content at later period. Superoxide dismutase (SOD) [60] and catalase (CAT) [61] were reported to play major roles in the defense against toxic ROS, and they were found to increase in the early phase of drought and decrease as the drought worsens [62]. So, SOD and CAT in pearl millet may respond quickly to drought signals. Simultaneously, the REC rose gradually from 0 h to 7 h no matter in CK or treatment groups. However, no significant difference between the CK and drought stress became apparent at 7 h, which also suggests that there could be some compounds that were produced to alleviate this situation.

### Data analysis of RNA-Seq

A total of 18 qualified cDNA libraries were separately constructed and used for RNA-Seq. The quality of RNA-Seq was decided based on the quality of sequencing and the correlations of biological replicates. In this study, the Q20 or Q30 exceeded 93% and the percentage of GC was greater than 53%. There are two sets of data that had low correlation with the other two biological replicates in the correlation analysis, we discarded them in other subsequent analysis. Furthermore, the FPKM values of 16 samples were assessed by Pearson correlation (R2) and Principal component analysis (PCA) (Supplemental Figure 1), which indicated that the quality of sequencing was high. Overall, the data of RNA-Seq is reliable and can be used to perform the additional analysis.

### Analysis of DEGs among drought stress and control conditions

**Fig. 2:**
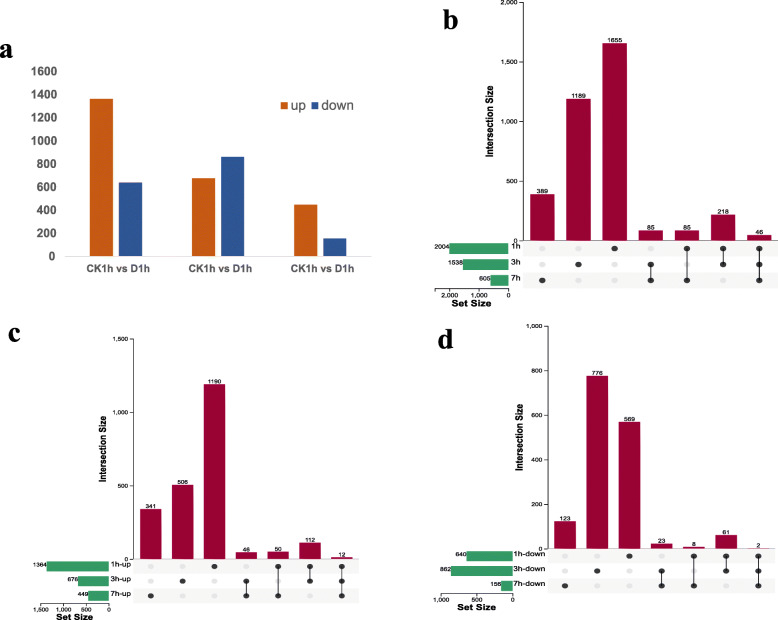
**a** The number of DEGs between CK and drought stress group at 1 h, 3 h and 7 h. **b** Upset diagram of all DEGs at three time points **c** Upset diagram of up-regulated DEGs at three time points **d** Upset diagram of down-regulated DEGs at three time points

To determine the DEGs involved in response to drought stress, three comparisons (total DEGs of three time points, up-regulated DEGs of three time points and down-regulated DEGs of three time points) were performed with a threshold of |log_2_ (FC)| ≥ 1 and *P* value ≤0.05 (Fig. 2). There were 2004 (1364 up-regulated and 640 down-regulated), 1538 (676 up-regulated and 862 down-regulated) and 605 (449 up-regulated and 156 down-regulated) genes that showed different levels of expression after 1 h, 3 h and 7 h drought treatment respectively (Fig. 2a, Supplemental Table 1). In addition, an upset [63] Venn analysis was performed for all the DEGs (Fig. 2b), up-regulated DEGs (Fig. 2c) and down-regulated DEGs (Fig. 2d). Twelve DEGs showed up-regulation at all three time points (Table 1). Alternatively, 1655, 1189 and 389 genes were differentially expressed specifically at 1 h, 3 h and 7 h of drought stress respectively (Fig. 2b). The number of DEGs decreased as the time of drought was extended.

**Table 1:** Description and expression of 12 up-regulated genes at all three time points

ID	Description
***i6_HQ_LWC_c101/f3p0/7152***	unclear
***i2_HQ_LWC_c28624/f2p1/2038***	diacylglycerol kinase
***i2_HQ_LWC_c118250/f16p0/2100***	NADP-dependent malic enzyme
***i2_LQ_LWC_c41668/f1p22/1915***	amino acid transporter
***i2_LQ_LWC_c131175/f1p1/2564***	phenylalanine/tyrosine ammonia-lyase-like
***i2_LQ_LWC_c13097/f1p0/2421***	plant cysteine oxidase 2-like
***i2_LQ_LWC_c34188/f1p2/2518***	unclear
***i1_HQ_LWC_c21568/f5p0/1463***	alcohol dehydrogenase (adh1C gene)
***i3_HQ_LWC_c32007/f19p0/3190***	zinc finger CCCH domain-containing protein
***i2_HQ_LWC_c122894/f9p1/2290***	unclear
***i1_HQ_LWC_c16500/f2p0/1894***	unclear
***i2_LQ_LWC_c81803/f1p19/2350***	ATP-dependent zinc metalloprotease FtsH 2

12 DEGs in table 1 are up-regulated at all three time points

The number of DEGs decreased from 1 h to 7 h, which had the same trend with the content of MDA under drought stress. This indicates that on exposure to drought stress a large number of ROS were produced and pearl millet immediately showed responses due to high drought resistance capability. Many genes were expressed, which resulted in the production of many proteins, such as enzymes respond to the sudden shock, and after that, the cells returned to a relatively balanced level. It could be a signal for plants that they were in a normal condition so in a short time, it was not essential to express many genes but just grew normally. This phenomenon also appeared in maize [64]. Both drought tolerant cultivar and drought sensitive cultivar maize faced drought stress at the seedling stage, and the number of DEGs in primary roots at 12 h was less than that at 0 h.

In our research, 12 genes exhibited up-regulation at all three time points, which could illustrate that they play some important roles in response to drought stress. A search of the annotation and sequence alignment resulted in a preliminary understanding of these up-regulated genes shown as Table 1 (The expression level of these genes was shown in Supplemental Figure 3). For these up-regulated genes, four of their functions were unclear, but the expression of *i6_HQ_LWC_c101/f3p0/7152*, *i2_LQ_LWC_c34188/f1p2/2518* and *i2_HQ_LWC_c122894/f9p1/2290* under drought treatment were 20-fold higher than those under normal conditions. Thus, it is essential to determine the function of these genes. Among other up-regulated genes, some genes were associated to show responses under environment stresses such as CCCH-type zinc fingers, zinc metalloprotease FtsH proteins, and alcohol dehydrogenase (ADH1 gene). CCCH-type zinc finger proteins are one group of zinc finger families, which typically contain 1–6 CCCH-type tandem zinc-binding motifs [65]. Many studies have suggested that the presence of CCCH is thought to be related to drought tolerance. For example, overexpression *PdC3H17* could confer tolerance to drought stress in *Populus* L. [66]. The CCCH family member *OsC3H47* was verified to promote drought tolerance and decrease ABA sensitivity in rice (*Oryza sativa*) [67]. In addition, the ABA pathway is a very important drought response pathway. Thus, CCCH may mediate the ABA pathway to render pearl millet more resistant to drought. Therefore, it could be one of reason why pearl millet is so tolerant to drought environment. The FtsH protein, encodes a Zn ^2+^- and ATP-dependent metalloprotease. It has been reported that *FtsH* is also related to stress adaptation [68, 69], but most of them were related to bacterial resistant. In our research, this gene was significantly up-regulated by approximately 20 times more than that in the CK group (Supplemental Figure 3). This suggests that research on this gene may lead to explore new insights in drought tolerance. Alcohol dehydrogenase is a key enzyme that can catalyze the reduction of acetaldehyde to ethanol using NADH as a reductant. In *Arabidopsis thaliana*, ADH1 confers both abiotic and biotic stress resistance [70]. These up-regulated genes should be targets of additional study because they may play an important role in the drought resistance of pearl millet.

### GO and KEGG enrichment analysis

A GO enrichment analysis of the DEGs at three time points was performed. Most of the DEGs in each time point were different than those in other time points, but the top five GO terms of three categories that they enriched were nearly identical (Supplemental Figure 2, Supplemental Tables 2, 3 and 4). In the ‘Biological process’ category, larger genes enriched in ‘metabolic process’, ‘cellular process’, ‘single-organism process’, ‘localization’ and ‘biological regulation’ (Supplemental Figure 2a). Fewer genes were enriched in ‘Cellular component’ than those in ‘Biological process’ and ‘Molecular function’ and the top five number of genes in the 3 h and 7 h treatments were related to ‘membrane’, ‘cell’, ‘cell part’, ‘membrane part’ and ‘macromolecular complex’, while for 1 h, there is no ‘macromolecular complex’ but ‘organelle’ (Supplemental Figure 2b). In the ‘Molecular function’ category, most of DEGs were enriched in ‘catalytic activity’, ‘binding’ and ‘transporter activity’ (Supplemental Figure 2c). At 1 h and 7 h after drought treatment, it was apparent that there were more up-regulated genes than down-regulated genes in each category and the situation was totally opposite to DEGs at 3 h after drought stress.

**Fig. 3:**
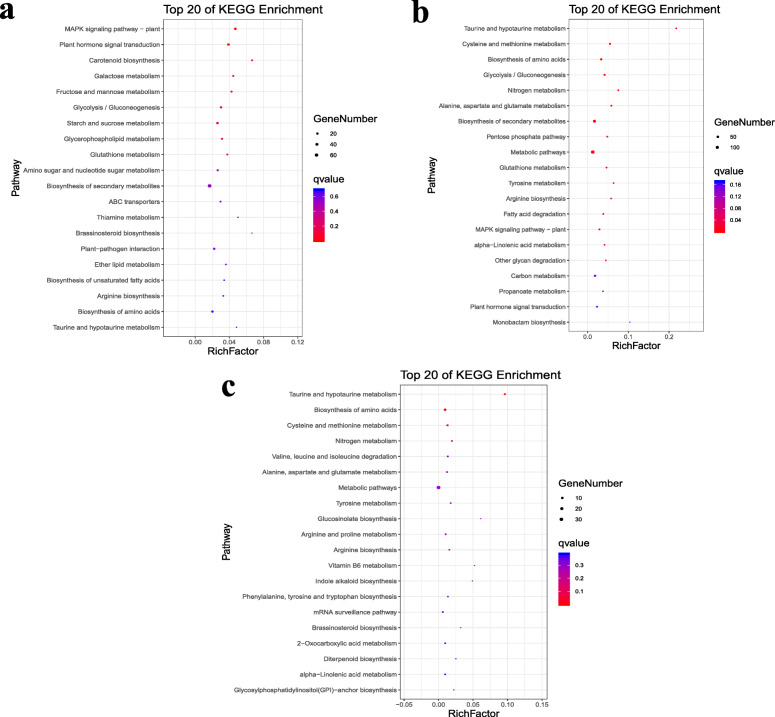
Analysis of DEGs that were differentially expressed between CK and drought stress. **a**, **b**, **c** KEGG analysis of DEGs specific to drought stress at 1 h, 3 h and 7 h

Simultaneously, the DEGs in each time point were also analyzed for their KEGG function (Fig. 3). At 1 h, the DEGs were significantly enriched into ‘MAPK signaling pathway – plant’, ‘Plant hormone signal transduction’ and ‘Galactose metabolism’ pathways (Fig. 3a, Supplemental Table 5). At 3 h after drought treatment, the DEGs were significantly enriched into 13 pathways and they were ‘Taurine and hypotaurine metabolism’, ‘Cysteine and methionine metabolism’, ‘Glycolysis / Gluconeogenesis’, ‘Nitrogen metabolism’, ‘Alanine, aspartate and glutamate metabolism’, ‘Biosynthesis of secondary metabolites’, ‘Pentose phosphate pathway’, ‘Metabolic pathways’, ‘Glutathione metabolism’, ‘Tyrosine metabolism’, ‘Arginine biosynthesis’ and ‘Fatty acid degradation’ (Fig. 3b, Supplemental Table 6), which has much more variation than those at in 1 h. There were just two pathways (‘Taurine and hypotaurine metabolism ‘and ‘Biosynthesis of amino acids’) in which the DEGs were significantly enriched after 7 h drought stress (Fig. 3c, Supplemental Table 7).

Plants can manage drought stress through the manipulation of some key physiological processes, such as respiration and antioxidant and hormonal metabolism [71]. In the enrichment analysis of GO term on ‘Biological process’, most of the DEGs were significantly enriched into metabolic processes. Respiration is a highly crucial metabolic process, which plays necessary roles in drought response. The rate of respiration is regulated by processes that use the respiratory products such as ATP, NADH and TCA cycle intermediates, which contribute to plant growth. Under drought stress, these processes will be affected and lead to a decline in the rate of respiration. Alternatively, higher respiration may arise because of oxidative phosphorylation, reducing the generation of ROS and preventing the accumulation of reductants. In addition, the activation of energy-intensive processes and increased respiratory rates, such as osmolyte synthesis and antioxidant metabolism, occur under drought conditions [72, 73]. Under drought treatment, ROS are generated owing to the metabolic perturbation of cells, and these molecules cause cell damage and death [74–76]. A very important adaptive mechanism will involve their effective scavenging. When these harmful substances produced in excessive amount, the antioxidant substrates such as carotenoids, and ascorbate, as well as a-tocopherol and antioxidant enzymes, such as SOD, CAT, APX and glutathione reductase, exist in cell organelles and the cytoplasm and play a crucial role in detoxifying these reactive species [77]. These important antioxidant enzymes are produced by some metabolic processes, so that when the plant suffers a shock of drought stress, an enormous number of metabolically related genes need to be expressed at a high level to respond to drought, particularly in drought tolerance species such as pearl millet. Furthermore, many DEGs were enriched into ‘membrane’ in ‘Cellular component’. This could be because ROS will attack the membranes of cells, and plants try to express related genes to repair them. Transcription factors (TFs) are also very important and play crucial role during this process. TFs act as switches and trigger the expression of an enormous number of stress-response genes that contribute to the stress phenotype [78]. To date, many TFs have been identified that are related to drought resistance and are members of the to bHLH, bZIP, HD-ZIP, AP2/ERF, MYB, EAR, NAC, NF-Y and ZPT2 families [71]. These TFs can bind with some specific sites or target genes and then regulate them. In our research, there were a large number of DEGs clustered into ‘binding’, which could be owing to the need of binding events between TFs and their important targets.

When subjected to drought stress, signal transduction in plants is very common. Mitogen-activated protein kinases (MAPK) phosphorylate proteins, which constitutes one of the main mechanisms of signal transduction in plants. Located in the cytoplasm, they consist of three types of enzymes (MAPK, MAPKK and MAPKKK) that form a signaling cascade from the stress sensor located on the plasma membrane to the regulation of gene expression in the nucleus. Transferring the MAPK to the nucleus activates transcription factors through phosphorylation [71, 79]. In our study, a large number of genes were located in the ‘MAPK signaling pathway- plant’ of KEGG enrichment analysis at 1 h and 3 h. This result showed that pearl millet positively responded to drought environment by enhancing signal transduction. At same time, we can find that few DEGs were enriched in ‘MAPK signaling pathway-plant’ at 7 h. This could be because as we discussed earlier, after drought shock, enormous numbers of genes were highly expressed and generated proteins to maintain the balance of cells by a series of biochemical reactions so that there was no need for so many genes to be expressed at a relative steady state.

Phytohormones are central factors that sense and signal various environmental conditions, such as drought stress [80]. When exposed to water deficits, ABA synthesized in the roots and translocated to leaves, where it led to stomatal closure that enabled the adaptation of plants to drought condition [81]. Previously published literature [82–84] indicates that the biosynthesis of ABA is strongly associated to drought stress response. Cytokinins are a type of negative regulatory factor for root growth and branching. The root-specific degradation of cytokinins could contribute to primary root growth and branching, which are induced by drought stress, andincrease drought tolerance in Arabidopsis [85]. Jasmonic acid (JA) and its metabolites, collectively known as jasmonates, originate from lipid oxidation pathways. Jasmonate signaling is associated with stress responses, including defense responses against biotic stressors, such as pathogens and insects, and also responses to abiotic stresses [80]..

### Weighted gene co-expression network analysis

**Fig. 4:**
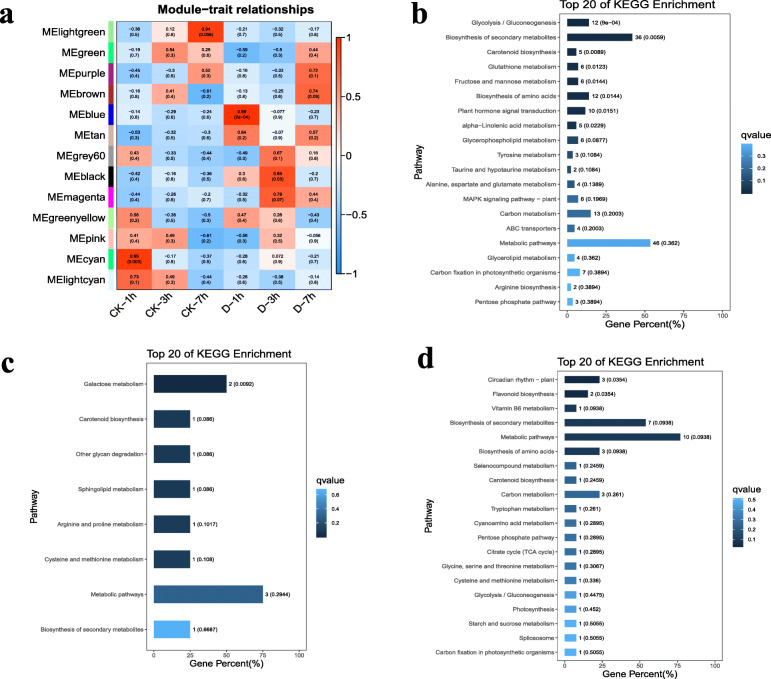
**a** A weighted correlation network analysis of genes at 6 groups. **b** KEGG analysis of MEblue module. **c** KEGG analysis of MEblack and MEmagenta. **d** KEGG analysis of MEpurple and MEbrown

The co-expression network of candidate DEGs was constructed using weighted gene co-expression network analysis (WGCNA). According to pairwise correlations and gene expression trends among all the samples, co-expression networks were constructed using a normalized FPKM value after RNA-Seq of all the DEGs from all samples using the WGCNA R package. The DEGs were clustered into 13 modules (lightgreen, green, purple, brown, blue, tan, grey60, black, magenta, yellow, pink, cyan and lightcyan) with high correlation values (Fig. 4b) and DEGS that could not gather into modules were abandoned. Notably, the MEblue (0.99), MEblack (0.85), as well as the MEmagenta (0.78) and MEpurple (0.72) and brown (0.74) modules, were highly correlated with drought at 1 h, 3 h and 7 h, respectively. In addition, sample clustering was also performed through their gene expression (Fig. 4a).

Furthermore, the DEGs in three modules that had the highest correlation with drought in each time point after drought treatment were analyzed using KEGG pathway analysis (Fig. 4). The number of DEGs in the module (MEblue) that had the highest correlation to drought stress for 1 h was 388, and they were significantly enriched into ‘Glycolysis / Gluconeogenesis’, ‘Biosynthesis of secondary metabolites’, ‘Carotenoid biosynthesis’, ‘Glutathione metabolism’, ‘Fructose and mannose metabolism’, ‘Biosynthesis of amino acids’, ‘Plant hormone signal transduction’ and ‘alpha-Linolenic acid metabolism’ pathways (Fig. 4b) and the DEGs that correlated with 3 h’ of drought stress (80) significantly clustered into only one pathway (‘Galactose metabolism’) (Fig. 4c). ‘Circadian rhythm – plant’ and ‘Flavonoid biosynthesis’ were two pathways, in which the DEGs related to 7 h’ water deficit (113) enriched into significantly (Fig. 4d).

As is shown in Fig. 4, some DEGs related to 1 h of drought stress (MEblue) were significantly clustered into ‘Plant hormone signal transduction’ and ‘MAPK signaling pathway-plant’ pathways, while not shown at 3 h and 7 h. Both pathways were identified as related to stress responses [78–80]. These results suggest that after 1 h of drought stress, or even earlier than 1 h, some important genes began to play roles in drought tolerance in the roots of pearl millet through a series of biochemical reactions. This process provides a certain degree of effect for cells to maintain homeostasis in a short period of time, which is consistent with the results of entire study.

### ABA signaling pathway

**Fig. 5:**
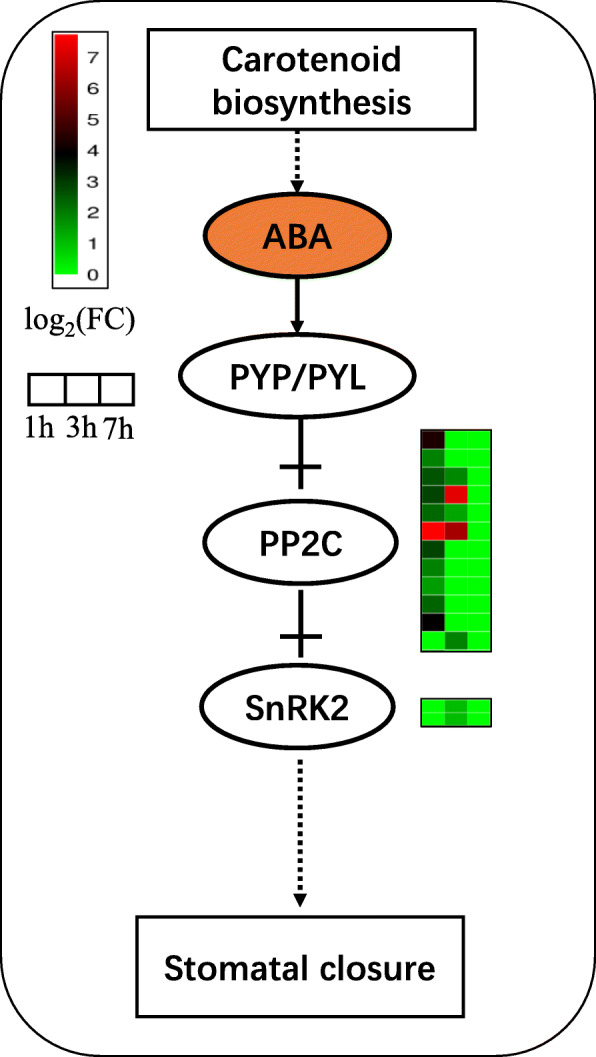
ABA signaling transduction pathway. Heatmaps represent the log_2_(FC) of genes at the corresponding time points

Nineteen genes were found to participate in the ABA signaling pathway in response to drought stress. Eleven PP2C-type protein phosphatases (PP2C) genes were differentially expressed under drought stress in pearl millet at 1 h, while there was only one that did not differ between the CK and drought stress group, increased under drought stress at 3 h and was restored to the same level as that in CK at 7 h. At 3 h, 4 out of 11 genes were still over expressed under drought compared with those of the CK. However, all 12 of these genes went down to the level of CK at 7 h. Besides, two SnRK2-type protein kinases (SnRK2s) displayed an up-regulated expression in the ABA signal transduction pathway at 3 h under a water deficit environment but were the same as CK at 1 h and 7 h (Fig. 5).

ABA signaling components are implicated in the regulation of guard cell ion channels [86]. The pyrabactin resistance (PYR)-like (PYL) family has been found to bind directly to ABA to function as receptors although many other gene families, such as the regulatory component of ABA receptor (RCAR) family, have also been identified as ABA receptors [87–90]. The PYL/PYR family has also been shown to connect with the other ABA regulators, such as PP2C SnRK2 [91] and directly inhibit the phosphatase activity of PP2Cs [92]. In addition, genetic evidence revealed that PP2Cs are negative regulators of ABA signaling [93–95]. Many studies have proved several SnRK2–PP2C interactions that clearly function in ABA signaling [96]. For example, the interaction of A-type PP2Cs with OST1 (an SnRK2-type kinases) contributed to ABA-induced stomatal closure by controlling the phosphorylation status and activity of SLAC1 anion channel in guard cells [97] and the activation of SnRK2-type kinases (ABA-dependent) results from the removal of inhibitory effect of PP2Cs [98]. This can be regulated by the binding of PYL/RCARs with PP2Cs, leading to the activation of SnRKs and de-repression of the signaling pathway [89–91, 99]. Overall, in response to drought or other stresses, the content of ABA in the plants increased to reach a level high, so that it bound to the PYL/RCAR-type receptors and improved the interaction between PYL/ RCAR and PP2Cs, which can activate SnRK2s, and in turn, interact with and phosphorylate SLAC1 and other channels that lead to stomatal closure [86, 97]. In this study, we could not find any PYP/PYL gene that was differentially expressed between the CK and drought group. However, thePP2C genes were expressed more highly under drought conditions. This could be because the PYP/PYL genes had finished their role of responding to drought before the sample collection time point (1 h), and they had returned to a normal status as we described before. In this situation, these PYP/PYL proteins still exist in the cell and stimulate the expression of PP2Cs, which inhibited the SnRK2 (1 h). As the time increased to 7 h, the level of ABA was decreased to the release of PYP/PYL. Thus, the expression of PP2C was suppressed, which caused an improvement in SnRK2. In this manner, they contributed to the closure of stomatal and enhanced drought tolerance of pearl millet.

Based on the results, we concluded that some earlier time points should be examined because even at 1 h, pearl millet has shown drought resistance in the root at transcriptome level, which should be a future research target. Moreover, some important genes may only show their regulatory role in drought tolerance at an earlier stage (before1 hour).

## Conclusion

In this study, we tested the physiological indexes (REC and MDA) of root of pearl millet at 0 h, 1 h, 3 h and 7 h. The levels of REC and MDA tended to increase and decrease, respectively, which indicated that pearl millet showed rapid response to drought stress. Simultaneously, according to the analysis of transcriptome, we found that the number of DEGs decreased from 1 h to 7 h, which had a similar trend with the change in content of MDA. At the same time, we found that among the 12 genes that were up-regulated at the three time points, some genes were found to be associated with drought stress responses in other species, such as *CCCH*, *ADH1* and *FtsH*. These genes may have a strong relationship to drought tolerance of pearl millet and need to be further explored. Besides, the DEGs at 1 h were enriched into ‘metabolic processes’, ‘MAPK signaling pathway’ and ‘plant hormone signal transduction’, which have been reported to be related to drought response. These results suggest that pearl millet can return to a steady state after a short time of drought treatment, because of some changes in gene expression as well as physiological and biochemical changes. Furthermore, some genes that participate in ABA signal transduction pathways, such as PP2C and SnRK2 were found to have changes in their level of exprssion, particularly their differential expression at 1 h and 3 h, indicating that pearl millet could take actions in response to drought stress before 1 h, and the ABA signal transduction pathway played an important role in drought tolerance in pearl millet. This study can provide a theoretical basis to enhance drought resistance in other plants.

## Supplementary Information

**Additional file 1: Supplemental Figure 1. S1a**. the Pearson correlation based on all expressed genes. **S1b**. principal component analysis (PCA) based on all expressed genes. **Supplemental Figure 2.** Analysis of DEGs that were differentialy expressed between CK and drought stress. **S2a, S2b, S2c**. GO analysis of DEGs specific to drought stress at 1h, 3h and 7h. **Supplemental Figure 3.** These 12 DEGs were up-regulated at all three time points, and the heatmap was generated by the log_2_(FC).

**Additional file 2: Supplemental Table 1.** DEGs at 7h after drought treatment

**Additional file 3: Supplemental Table 2.** GO Enrichment at 1h

**Additional file 4: Supplemental Table 3.** GO Enrichment at 3h

**Additional file 5: Supplemental Table 4.** GO Enrichment at 7h

**Additional file 6: Supplemental Table 5.** KEGG enrichment at 1h

**Additional file 7: Supplemental Table 6.** KEGG enrichment at 3h

**Additional file 8: Supplemental Table 7.** KEGG enrichment at 7h

## Data Availability

The datasets supporting the conclusions of this article are included within the article (and its supplemental files). RNA-seq database for pearl millet could download from NCBI under the accession number PRJNA688001 (https://www.ncbi.nlm.nih.gov/sra/PRJNA688001), and the data will be shared on reasonable request of the corresponding author.
