# Supplementary material for: Research on the drought tolerance mechanism of Pennisetum glaucum (L.) in the root during the seedling stage
Source: BMC Genomics. 2021 Jul 23;22:568. doi: 10.1186/s12864-021-07888-5 (PMC8305952; doi:10.1186/s12864-021-07888-5)
Supplement: Supplementary file 1 — Additional file 1: Supplemental Figure 1. S1a. the Pearson correlation based on all expressed genes. S1b. principal component analysis (PCA) based on all expressed genes. Supplemental Figure 2. Analysis of DEGs that were differentialy expressed between CK and drought stress. S2a, S2b, S2c. GO analysis of DEGs specific to drought stress at 1h, 3h and 7h. Supplemental Figure 3. These 12 DEGs were up-regulated at all three time points, and the heatmap was generated by the log2(FC). [file 12864_2021_7888_MOESM1_ESM.docx]

**Research on the drought tolerance mechanism of *Pennisetum glaucum* (L.) in the root during the seedling stage**

**Ailing Zhang^1^*, Yang Ji^2^*, Min Sun^1^*, Chuang Lin^1^*, Puding Zhou^1^, Juncai Ren^3^, Dan Luo^1^, Xiaoshan Wang^1^, Encong Ma^1^, Xinquan Zhang^1^, Guangyan Feng^1^, Gang Nie^1^, Linkai Huang^1^****

^1^College of Grassland Science and Technology, Sichuan Agricultural University, Chengdu 611130, China

^2^Sichuan Animal Science Academy, Chengdu 610066, China

^3^College of Animal Science and Technology, Rongchang Campus, Southwest University, Chongqing, 402460, China

*****Ailing Zhang, Yang Ji, Min Sun and Chuang Lin contributed equally to this work

******Corresponding Author: [huanglinkai@sicau.edu.cn](mailto:huanglinkai@sicau.edu.cn)

**S1a**


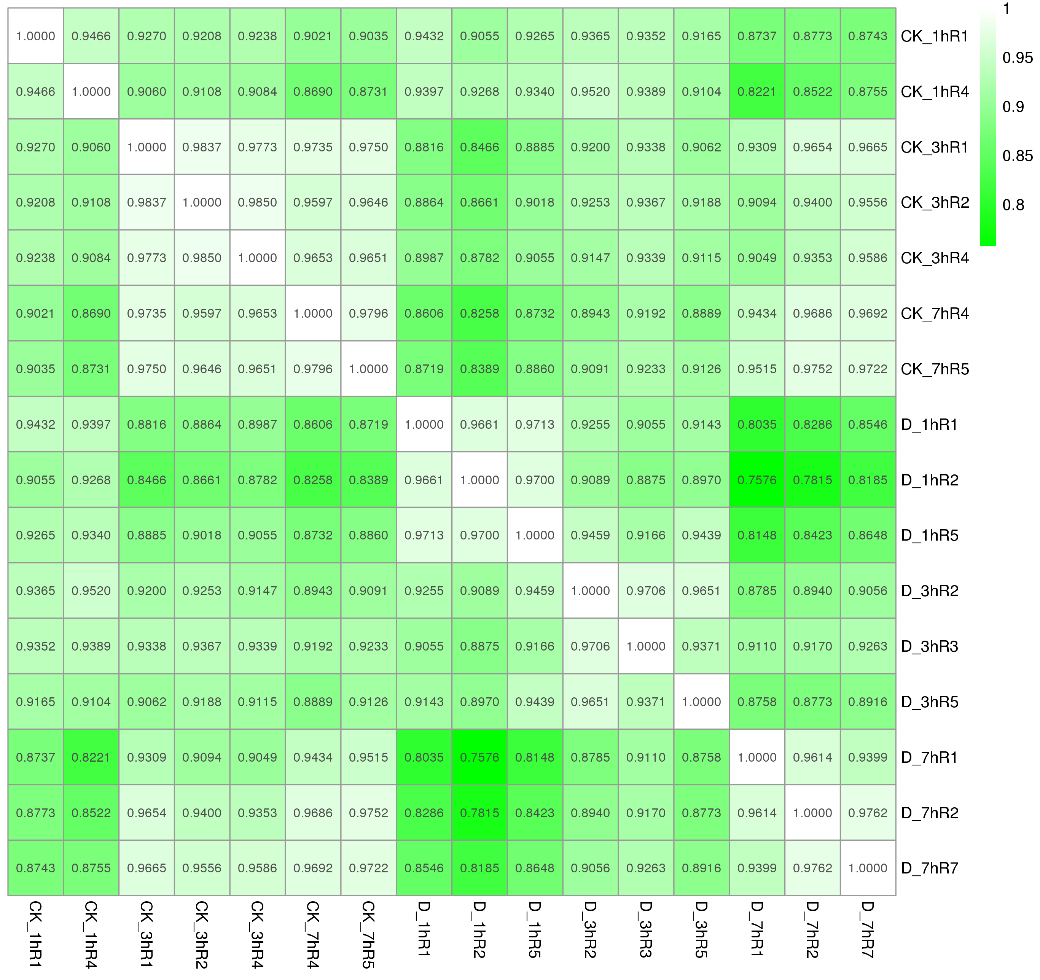


**S1b**


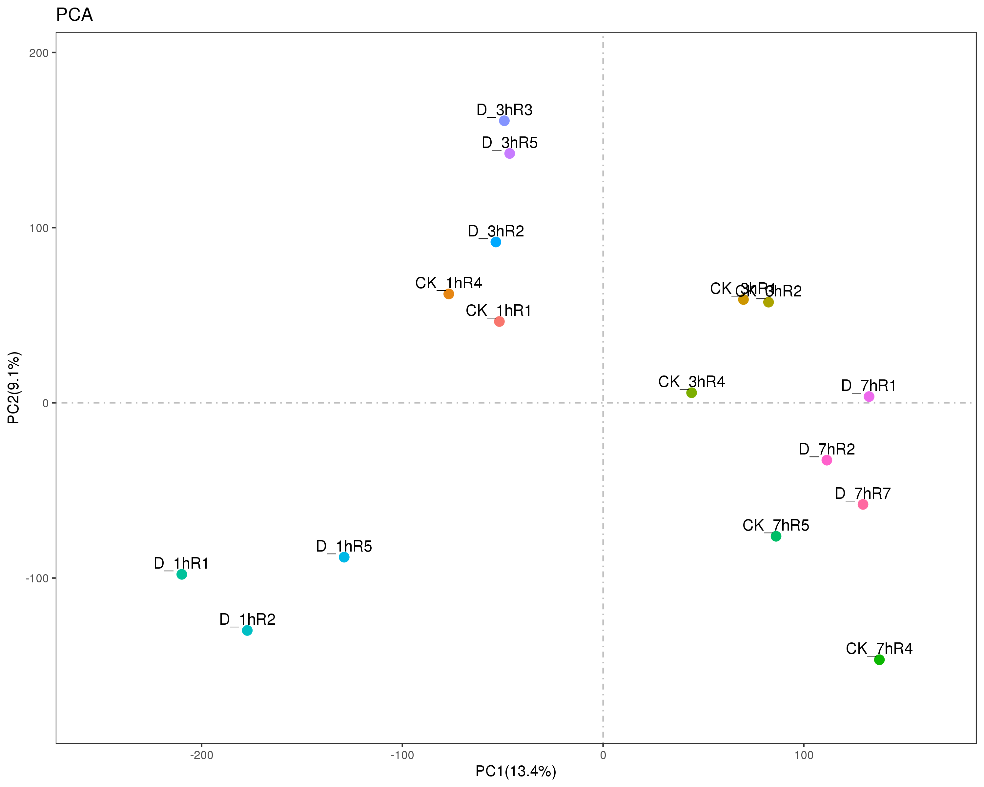


**Supplemental Figure 1**. **S1a**. the Pearson correlation based on all expressed genes. **S1b**. principal component analysis (PCA) based on all expressed genes.


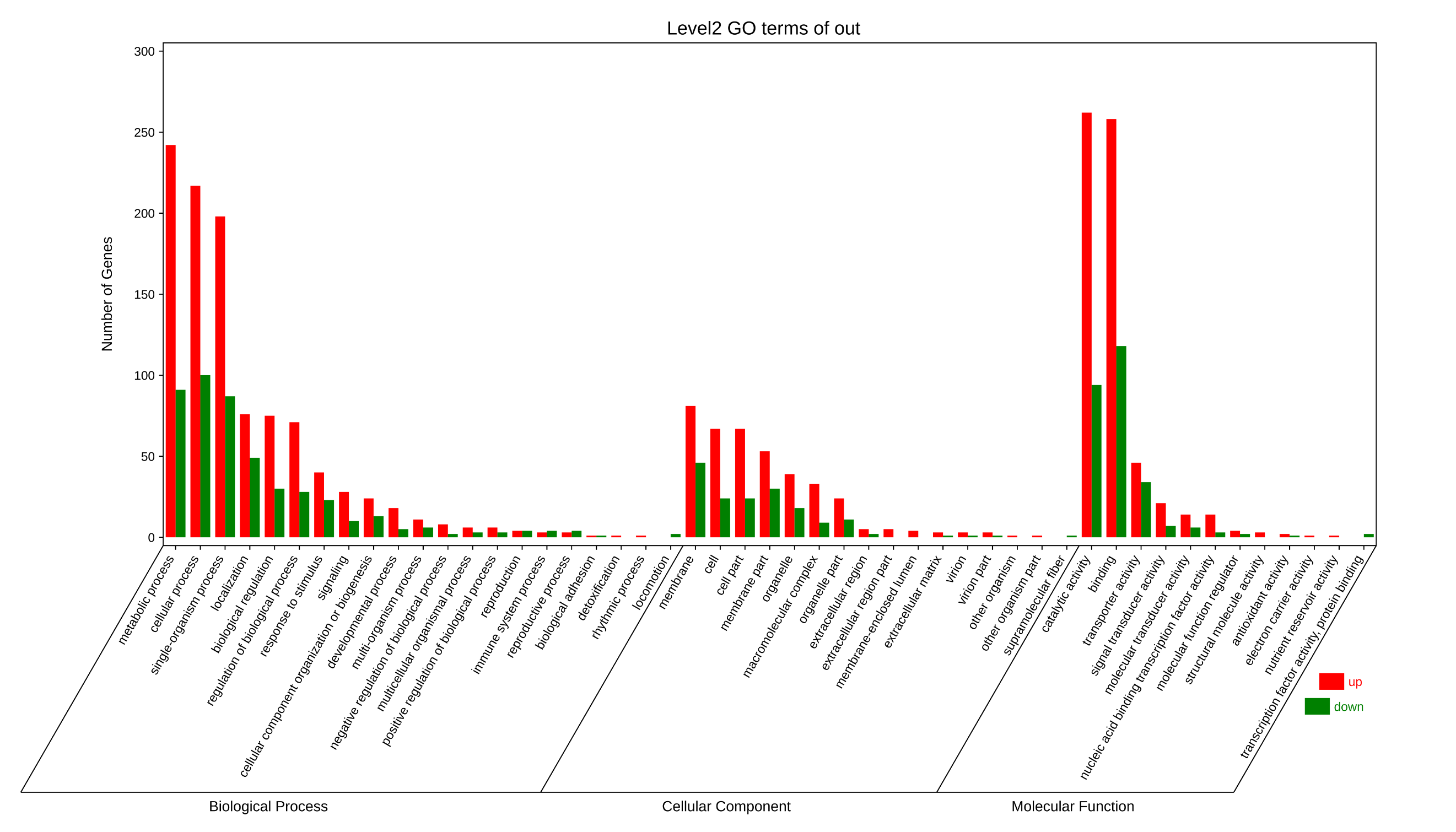


**S2a**

**S2b**


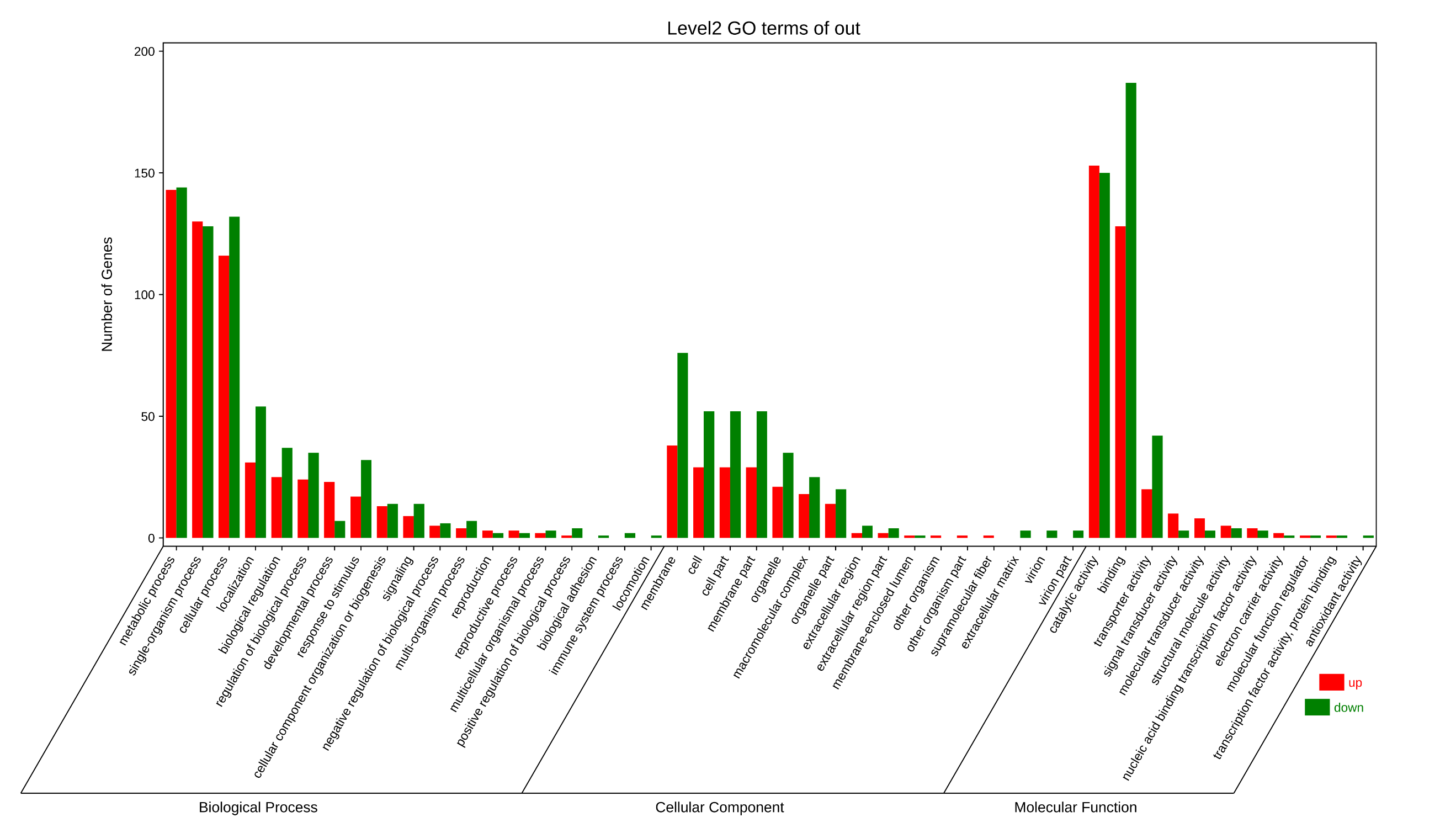


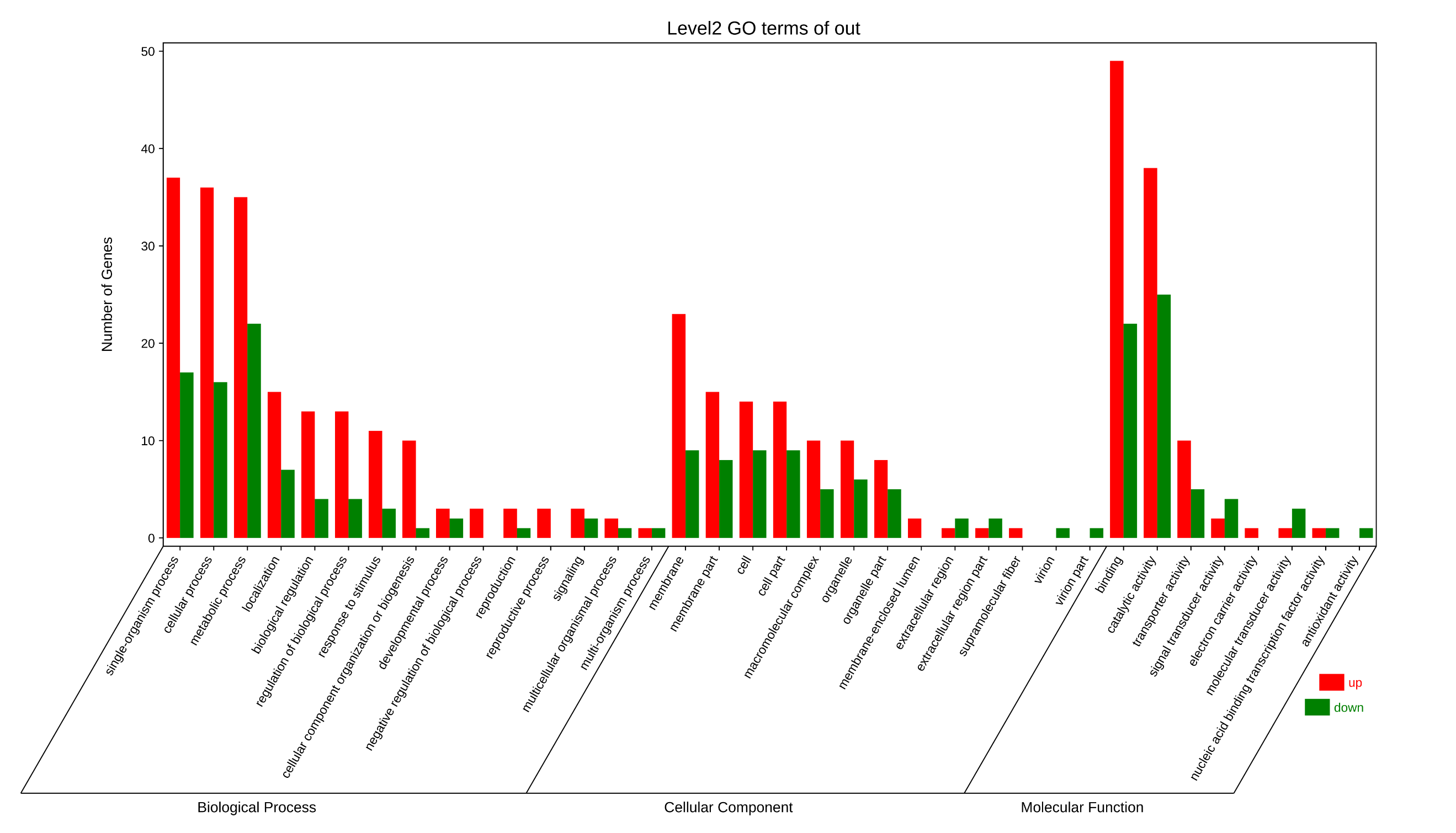


**S2c**

**Supplemental Figure 2**. Analysis of DEGs that were differentialy expressed between CK and drought stress. **S2a, S2b, S2c**. GO analysis of DEGs specific to drought stress at 1h, 3h and 7h.

**
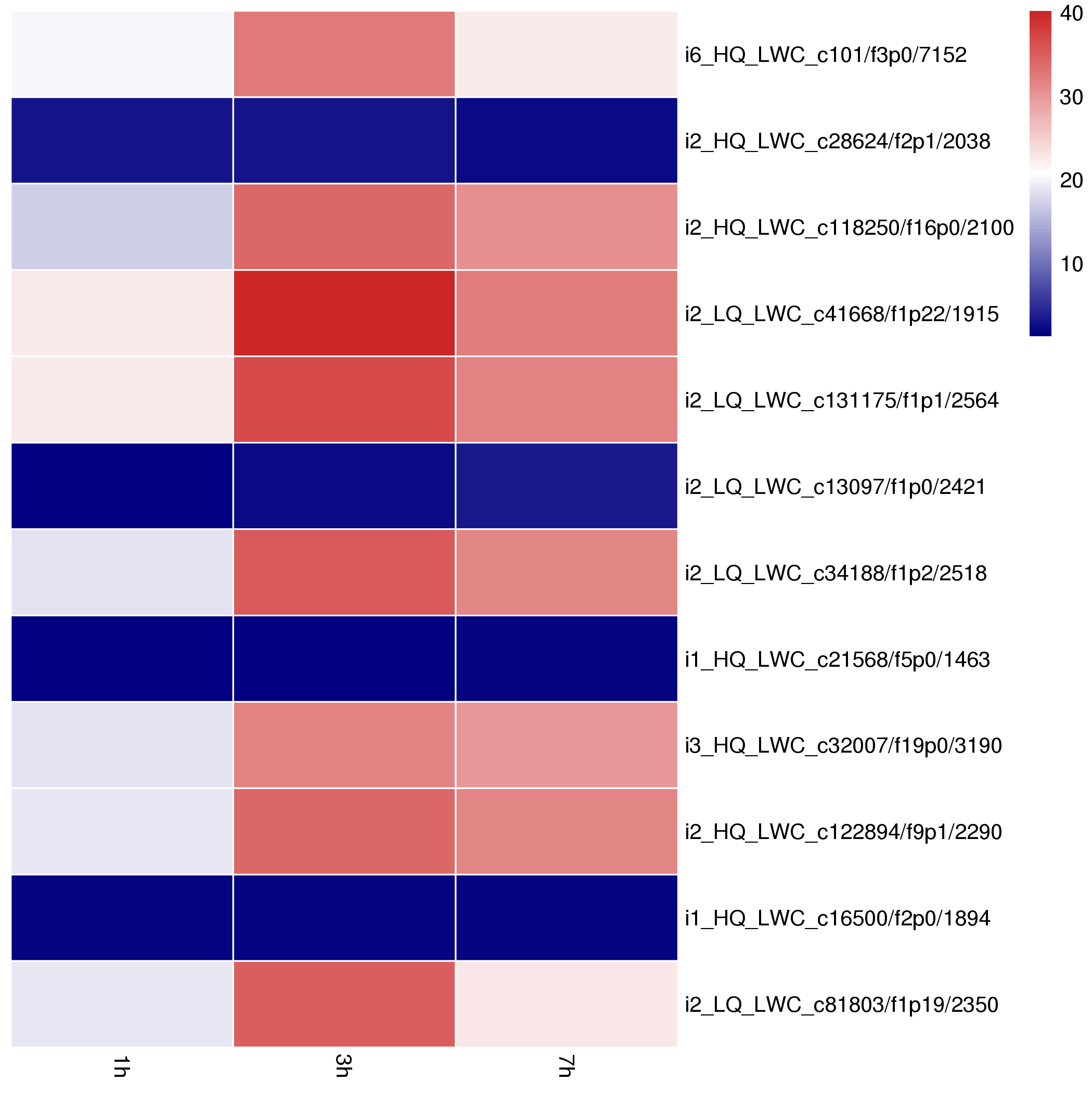
**

**Supplemental Figure 3**. These 12 DEGs were up-regulated at all three time points, and the heatmap was generated by the log_2_(FC).
